# Effectiveness of taxanes following nivolumab in patients with advanced esophageal squamous cell carcinoma: a retrospective chart review of patients in ATTRACTION-3

**DOI:** 10.1007/s10388-022-00972-z

**Published:** 2022-12-23

**Authors:** Keisho Chin, Shun Yamamoto, Masanobu Takahashi, Shigenori Kadowaki, Yutaro Kubota, Yusuke Amanuma, Morihito Okada, Mitsuro Kanda, Yasue Kimura, Yuhiko Nogi, Yuko Arimitsu, Yuko Kitagawa

**Affiliations:** 1 Department of Gastroenterological Chemotherapy, Cancer Institute Hospital, Japanese Foundation for Cancer Research, 3-8-31, Ariake, Koto, Tokyo, 135-8550 Japan; 2 Department of Head and Neck, Esophageal Medical Oncology, National Cancer Center Hospital, Tokyo, Japan; 3 Department of Medical Oncology, Tohoku University Hospital, Sendai, Japan; 4 Department of Clinical Oncology, Aichi Cancer Center Hospital, Nagoya, Japan; 5 Division of Medical Oncology, Department of Medicine, Showa University School of Medicine, Tokyo, Japan; 6 Department of Clinical Trial Promotion, Chiba Cancer Center, Chiba, Japan; 7 Department of Surgical Oncology, Hiroshima University, Hiroshima, Japan; 8 Department of Gastroenterological Surgery, Nagoya University Graduate School of Medicine, Nagoya, Japan; 9 Department of Surgery and Science, Graduate School of Medical Sciences, Kyushu University, Fukuoka, Japan; 10 Oncology Clinical Development, Bristol Myers Squibb, Tokyo, Japan; 11 Department of Oncology, ONO Pharmaceutical Co. Ltd., Osaka, Japan; 12 Department of Surgery, Keio University School of Medicine, Tokyo, Japan

**Keywords:** Esophageal squamous cell carcinoma, Nivolumab, Taxane

## Abstract

**Background:**

The phase III ATTRACTION-3 study showed that second-line nivolumab monotherapy for advanced esophageal squamous cell carcinoma prolonged overall survival (OS) but did not improve progression-free survival (PFS). Subsequent systemic therapy after discontinuing nivolumab may affect these outcomes. To test this possibility, we evaluated the outcomes of treatment with taxanes after nivolumab in ATTRACTION-3.

**Methods:**

We reviewed the charts of Japanese patients who had discontinued second-line nivolumab in ATTRACTION-3 and started subsequent third-line taxanes between January 7, 2016, and November 12, 2018. The primary endpoint was objective response rate (ORR) to third-line taxanes.

**Results:**

Of the 75 patients included in this study, 54 (72%), 18 (24%), and 3 (4%) patients received either paclitaxel, docetaxel, or combination therapy comprising docetaxel, cisplatin, and 5-fluorouracil, respectively. The ORR in the overall, paclitaxel, and docetaxel groups was 29.6%, 36.5%, and 12.5%, respectively; these numbers were comparable to those (20–44%) in patients receiving taxanes as first- and second-line therapy. The median OS in the overall, paclitaxel, and docetaxel groups was 9.9, 9.9, and 9.3 months, respectively, whereas the corresponding median PFS was 4.9, 4.7 and 6.5 months, respectively. Treatment-related adverse events were observed in 65 (87%) patients, of which grade 3–4 occurred in 37 (49%) patients.

**Conclusions:**

Favorable effectiveness and safety profile of taxanes following second-line nivolumab was observed in Japanese patients with advanced esophageal squamous cell carcinoma. When a patient with advanced esophageal squamous cell carcinoma receiving nivolumab becomes refractory or intolerant, subsequent taxane treatment may be a promising option.

**Supplementary Information:**

The online version contains supplementary material available at 10.1007/s10388-022-00972-z.

## Introduction

Esophageal cancer is the seventh most common cancer and the sixth leading cause of cancer death worldwide [1]. Advanced esophageal cancer has a poor prognosis with a 5-year survival rate as low as 5–10% [2].

Monotherapy with immune checkpoint inhibitors (ICIs) including anti-programmed cell death-1 (PD-1) antibodies, nivolumab and pembrolizumab, has recently been introduced as a standard second-line treatment for advanced esophageal cancer [3, 4]. A phase III ATTRACTION-3 study demonstrated that second-line nivolumab monotherapy improved overall survival (OS) as the primary endpoint, with a median of 10.9 compared to that of 8.5 months with chemotherapy [hazard ratio (HR) 0.79; 95% confidence interval (CI) 0.64–0.97] in patients with advanced esophageal squamous cell carcinoma and a 3-year OS rate of 15.3% and 8.7%, respectively [3, 5]. The duration of response to nivolumab (median, 6.9 months) was longer than that to chemotherapy (median, 3.9 months). On the other hand, progression-free survival (PFS) was comparable between patients treated with nivolumab and chemotherapy, with a median of 1.7 and 3.4 months, respectively (HR 1.07; 95% CI 0.87–1.33). The objective response rate (ORR) was also comparable (19% and 22%, respectively). These trends in OS, PFS, and ORR were also observed in the Japanese subpopulation (median OS, 13.2 vs. 8.7 months; median PFS, 2.7 vs. 3.8 months; ORR, 22% vs. 22%, respectively) [6].

Before the introduction of ICIs, no drug had demonstrated OS benefit in the second-line treatment for advanced esophageal cancer. Monotherapy with taxanes such as docetaxel and paclitaxel has been often used, although their limited efficacy was only evaluated in single-arm phase II studies [7–9]. After the standard second-line treatment with ICIs was established, the use of taxanes was shifted to third-line treatment. Several studies have demonstrated that ICI therapy may enhance the anti-tumor activity of subsequent chemotherapy for multiple cancers including gastric cancer, squamous cell carcinoma of the head and neck, and non-small cell lung cancer [10–13]. These results suggest that the anti-tumor activity of chemotherapy for esophageal cancer can also be enhanced by preceding ICI therapy, which may explain the OS prolongation without PFS improvement by second-line nivolumab in ATTRACTION-3 [3]. To test this possibility, the impact of ICIs on the effectiveness of subsequent therapy in advanced esophageal cancer should be evaluated.

Of the 136 Japanese patients in the nivolumab group in ATTRACTION-3, 80 (59%) patients received systemic chemotherapy after nivolumab discontinuation [6]. Because 77 out of these 80 patients received taxanes as the third-line treatment, the present chart review study aimed to retrospectively evaluate the effectiveness and safety of subsequent taxanes after nivolumab discontinuation in patients with advanced esophageal squamous cell carcinoma enrolled in ATTRACTION-3.

## Methods

### Study design and patients

ATTRACTION-3 is an international, randomized, open-label, phase III study evaluating nivolumab over investigator’s choice of chemotherapy (paclitaxel or docetaxel). Patients were enrolled in ATTRACTION-3 between January 7, 2016, and May 25, 2017, if they had unresectable esophageal cancer that was pathologically confirmed as squamous or adenosquamous cell carcinoma, and were refractory or intolerant to fluoropyrimidine-based and platinum-based chemotherapy. Nivolumab 240 mg was administered every 2 weeks until disease progression or unacceptable toxicity. The treatment was permitted to continue beyond initial disease progression at the discretion of the investigator.

The present retrospective chart review study enrolled Japanese patients who discontinued the second-line nivolumab treatment in ATTRACTION-3 and started subsequent third-line chemotherapy with taxanes by November 12, 2018. The date of data cutoff was December 31, 2020.

The present study was conducted in accordance with the Declaration of Helsinki and the Japanese Ethical Guidelines for Medical Research Involving Human Subjects. The study protocol was approved by the ethical review board of each institute. All patients or their legal representatives provided written informed consent and were given the opportunity to opt out.

### Assessments

The present study primarily evaluated ORR to third-line taxanes. Tumor responses were assessed by each investigator according to the Response Evaluation Criteria in Solid Tumors, version 1.1 [14]. Tumor responses included complete response (CR), partial response (PR), stable disease (SD), and progressive disease (PD). ORR was defined as the proportion of patients who achieved confirmed or unconfirmed CR or PR. Secondary endpoints included the disease control rate (DCR), defined as the proportion of patients with CR, PR, or SD; OS and PFS from the start of the third-line taxanes; and safety. Adverse events (AEs) were categorized using the Medical Dictionary for Regulatory Activities, Japanese version 23.0, and were graded by the Common Terminology Criteria for Adverse Events version 4.0.

### Statistical analysis

The 95% CI for ORR was calculated using the Clopper–Pearson method. OS and PFS were analyzed using the Kaplan–Meier method. The 95% CIs for their median and for rates at a specific time point were calculated using the Brookmeyer and Crowley method and the Greenwood’s formula, respectively. To identify patient subpopulation associated with high ORR, OS, and PFS, univariate logistic regression analysis for ORR and univariate and multivariate COX regression analyses for OS and PFS were conducted; independent variables included patient demographics and baseline characteristics that were considered to be associated with poor prognosis of esophageal cancer and to be useful for considering effectiveness of taxanes after nivolumab treatment. All statistical analyses were conducted using SAS version 9.4 (SAS Institute Japan Ltd., Tokyo, Japan).

## Results

Of the 77 Japanese patients who were receiving third-line treatment with taxanes after discontinuation of nivolumab in ATTRACTION-3, 75 patients provided informed consent for this chart review. Their median age was 65 years, and 81% patients were men (Table 1). The proportion of patients with the Eastern Cooperative Oncology Group performance status (ECOG PS) 0, 1, and 2 or higher was 43%, 43%, and 9%, respectively. Overall, 58 (77%) patients had lymph node metastasis, 20 (27%) had liver metastasis, and 32 (43%) had lung metastasis. The reason for discontinuation of the second-line nivolumab therapy was PD in 65 (87%) patients, AEs in 7 (9%), effectiveness in 2 (3%), and unknown in 1 (1%).

**Table 1:** Patient baseline characteristics at the start of the taxane therapy after discontinuing nivolumab (*n* = 75)

Characteristics	*n* (%)^a^
Age	
Median (range)—years	65 (42–81)
65 years or older	41 (55)
Men	61 (81)
ECOG PS	
0	32 (43)
1	32 (43)
2 or higher	7 (9)
Unknown	4 (5)
Primary lesion	
Yes	42 (56)
No	28 (37)
Unknown	5 (7)
Lymph node metastasis	
Yes	58 (77)
No	15 (20)
Unknown	2 (3)
Number of lymph node metastases	
1	13 (17)
2–4	17 (23)
5 or more	26 (35)
Unknown	2 (3)
Liver metastasis^b^	20 (27)
Lung metastasis^c^	32 (43)
Duration of nivolumab therapy	
Median (range)—months	2.8 (0.0–12.5)
Best overall response to nivolumab therapy^d^	
Complete response	0
Partial response	15 (21)
Stable disease	29 (40)
Progressive disease	27 (38)
Not evaluable	1 (1)
Reason for nivolumab discontinuation	
Progression of primary disease	65 (87)
Adverse events	7 (9)
Effectiveness	2 (3)
Others	1 (1)

*ECOG PS* eastern cooperative oncology group performance status

^a^Number (percent) of patients, unless otherwise noted

^b^Unknown, 2 patients

^c^Unknown, 3 patients

^d^A total of 72 patients had target lesion measurements before nivolumab therapy

As the third-line treatment, 54 (72%) patients received paclitaxel, 18 (24%) received docetaxel, and 3 (4%) received a combination therapy with docetaxel, cisplatin, and 5-fluorouracil (DCF therapy). Patient characteristics were similar between patients receiving paclitaxel and docetaxel except for the proportion of patients aged 65 years or older (paclitaxel, 67%; docetaxel, 22%), with ECOG PS 2 or higher (paclitaxel, 13%; docetaxel, 0%), and with liver metastasis (paclitaxel, 33%; docetaxel, 11%) (Supplementary Table 1). The response evaluable population comprised 71 patients in total, 52 receiving paclitaxel, 16 receiving docetaxel, and 3 receiving DCF therapy. A swimmer plot for the response evaluable population illustrates the treatment course of each patient (Supplementary Fig. 1). The median duration between nivolumab discontinuation and the start of taxanes was 0.8 (95% CI 0.7–1.0) months.

### Effectiveness

The ORR to third-line taxanes, the primary endpoint, was 29.6% (95% CI 19.3–41.6%). The number of patients with a best overall response of CR, PR, SD, and PD was 0, 21 (30%), 27 (38%), and 15 (21%), yielding a DCR of 67.6%. The ORR by taxane regimen was 36.5% (95% CI 23.6–51.0%) for paclitaxel, 12.5% (95% CI 1.6–38.3%) for docetaxel, and 0% (95% CI 0–70.8%) for DCF therapy. The univariate logistic regression analysis showed that no factors, including the efficacy of the preceding nivolumab therapy, significantly influenced the ORR to the third-line taxanes (Supplementary Table 2).

**Fig. 1:**
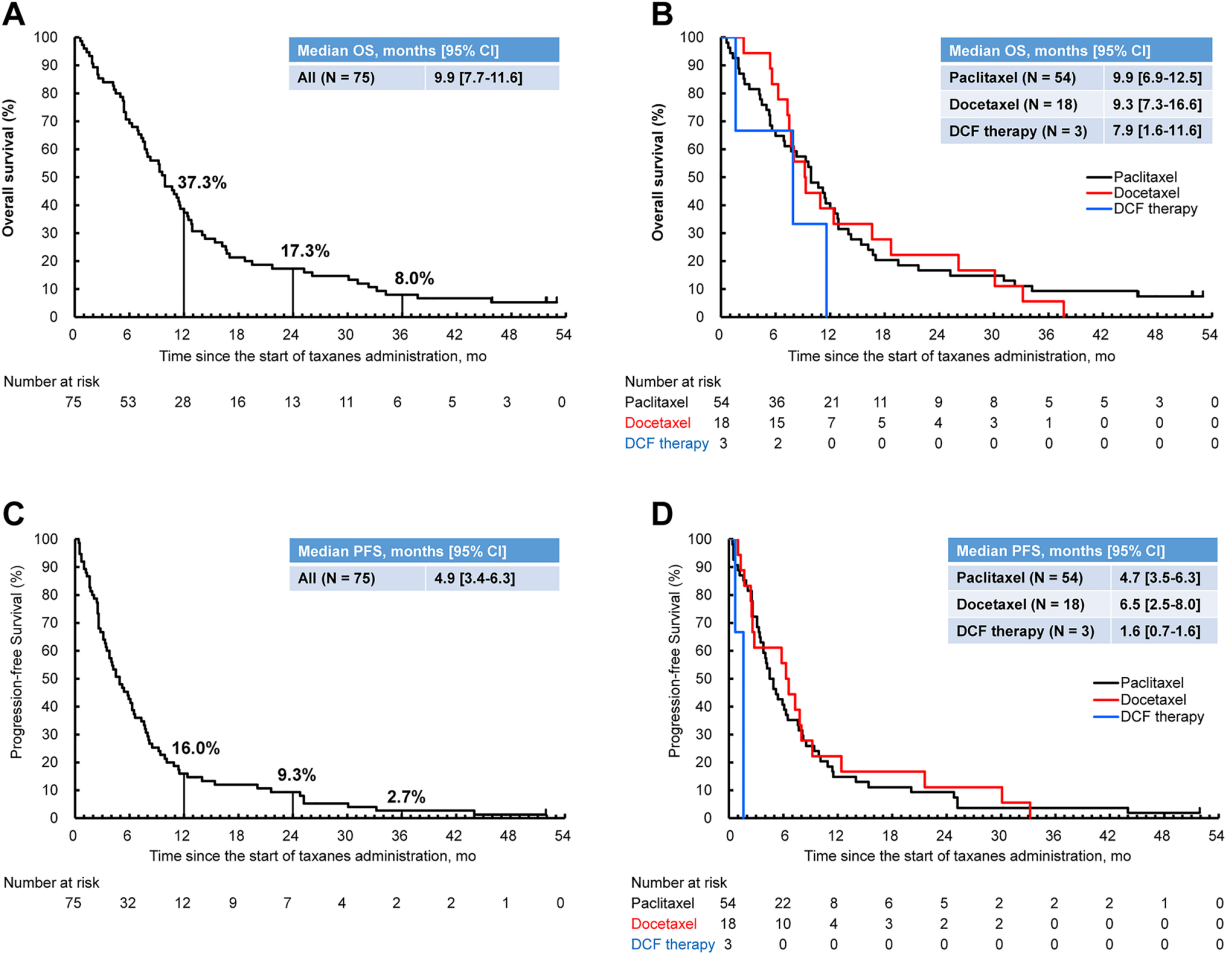
OS and PFS. The Kaplan–Meier curves of OS after the start of the taxane treatment in all patients (**A**) and in patients treated with each taxane regimen (**B**). The Kaplan–Meier curves of PFS after the start of the taxane treatment in all patients (**C**) and in patients treated with each taxane regimen (**D**). *DCF* docetaxel, cisplatin, and 5-fluorouracil, *mo* months, *OS* overall survival, *PFS* progression-free survival

The median OS was 9.9 (95% CI 7.7–11.6) months (Fig. 1A), and that in patients by taxane regimen was 9.9 (95% CI 6.9–12.5) months for paclitaxel, 9.3 (95% CI 7.3–16.6) months for docetaxel, and 7.9 (95% CI 1.6–11.6) months for DCF therapy (Fig. 1B). The multivariate analysis (Supplementary Table 3) showed that patients with lung metastasis had long median OS compared with those without lung metastasis (adjusted HR 0.46; 95% CI 0.23–0.94), patients with ≥ 5 metastatic sites had short median OS compared with those with 1–4 metastatic sites (adjusted HR 2.46; 95% CI 1.17–5.18), and patients with ECOG PS ≥ 2 had short median OS compared with those with ECOG PS 0 (adjusted HR 5.63; 95% CI 1.96–16.19). We noticed a significant imbalance in several background factors, including the number of lymph node metastases (*P* = 0.0359), between patients with and without lung metastasis (Supplementary Table 4).

The median PFS was 4.9 (95% CI 3.4–6.3) months (Fig. 1C), and that in patients by taxane regimen was 4.7 (95% CI 3.5–6.3) months for paclitaxel, 6.5 (95% CI 2.5–8.0) months for docetaxel, and 1.6 (95% CI 0.7–1.6) months for DCF therapy (Fig. 1D). The multivariate analysis (Supplementary Table 5) showed that, consistent with the results for OS, patients with lung metastasis had long median PFS compared with those without lung metastasis (adjusted HR 0.47; 95% CI 0.23–0.94), patients with ≥ 5 metastatic sites had short median PFS compared with those with 1–4 metastatic sites (adjusted HR 3.10; 95% CI 1.52–6.32), and patients with ECOG PS ≥ 2 had short median PFS compared with those with ECOG PS 0 (adjusted HR 3.32; 95% CI 1.21–9.09).

### Safety

Any AEs related to third-line taxanes [treatment-related AEs (TRAEs)] were observed in 65 (87%) patients, and grade 3–4 TRAEs occurred in 37 (49%) patients (Table 2). No patients died due to TRAEs. The most common TRAEs were neutropenia (37%), malaise (35%), peripheral neuropathy (31%), leukopenia (29%), alopecia (28%), nausea (25%), decreased appetite (24%), and anemia (20%).

**Table 2:** Treatment-related adverse reactions observed in ≥ 5% patients

TRAEs	Any grade	Grade 3–4
Any	65 (87)	37 (49)
Neutropenia	28 (37)	23 (31)
Malaise	26 (35)	0
Neuropathy peripheral	23 (31)	3 (4)
Leukopenia	22 (29)	12 (16)
Alopecia	21 (28)	0
Nausea	19 (25)	2 (3)
Decreased appetite	18 (24)	0
Anemia	15 (20)	7 (9)
Constipation	9 (12)	1 (1)
Diarrhea	9 (12)	0
Peripheral sensory neuropathy	9 (12)	2 (3)
Fatigue	8 (11)	1 (1)
Stomatitis	8 (11)	1 (1)
Thrombocytopenia	7 (9)	2 (3)
Edema peripheral	6 (8)	0
Febrile neutropenia	5 (7)	5 (7)
Pruritus	5 (7)	0
Hypoalbuminemia	4 (5)	0
Myalgia	4 (5)	0
Pyrexia	4 (5)	0
Rash	4 (5)	0

Data is represented as number (percent) of patients

*TRAE* treatment-related adverse event

## Discussion

In ATTRACTION-3, nivolumab as second-line therapy for advanced esophageal cancer showed benefit in OS but not in PFS [3]. This chart review study revealed that taxanes as third-line therapy after nivolumab discontinuation in Japanese patients enrolled in ATTRACTION-3 yielded an ORR of 29.6%, which is more favorable compared to the 22.2% in Japanese patients treated with paclitaxel or docetaxel as second-line therapy in ATTRACTION-3 [6]. The median OS of the third-line taxanes was 9.9 months, which is also comparable to that from the second-line taxanes (9.4 months). Benefit from the third-line taxanes was consistently observed across patients with and without response to the preceding nivolumab treatment and irrespective of the reasons for nivolumab discontinuation. These results suggest that subsequent taxane treatment may contribute to the overall survival of second-line nivolumab in ATTRACTION-3.

The ORR, median PFS, and median OS of the third-line taxanes following nivolumab were 29.6%, 4.9 months, and 9.9 months, respectively, in the present study. While the efficacy of third-line chemotherapy for advanced esophageal cancer has not been well demonstrated, the benefit from the third-line taxanes observed in the present study would favorably compare to that from earlier lines. A phase II study of docetaxel as first- or second-line treatment for metastatic esophageal cancer showed that the ORR was 20%, the median duration of response was 4.7 months, and the median OS was 8.1 months [8]. The ORR dropped to 16% when the treatment was restricted to second-line. A phase II study of paclitaxel in patients with advanced esophageal cancer who had received at least one platinum-based chemotherapy showed that the ORR was 44.2%, the median duration of response was 4.8 months, and the median OS was 10.4 months [7]; the observed high ORR may be attributed to the high dose (100 mg/m^2^) of paclitaxel. Multivariate analysis showed that both median OS and PFS were longer in patients with lung metastasis than in those without lung metastasis. The proportion of patients with 4 or ≥ 5 lymph node metastasis was higher in those without lung metastasis (4, 5%; ≥ 5, 38%) than in those with lung metastasis (4, 0%; ≥ 5, 19%). As a previous study suggested that the number of lymph node metastases is associated with poor prognosis [15], this imbalance in the number of lymph node metastases may have contributed to the difference in the OS and PFS between patients without and with lung metastasis.

A randomized phase II study for esophageal cancer refractory to fluoropyrimidine- and platinum-based chemotherapy demonstrated a favorable efficacy of paclitaxel compared to docetaxel; in patients treated with paclitaxel vs. docetaxel, the ORR was 25.6% vs. 7.7%, the median PFS was 4.4 vs. 2.1 months, and the median OS was 8.8 vs. 7.3 months [16]. The relatively favorable ORR was also observed in patients treated with third-line paclitaxel compared to third-line docetaxel in the present study, whereas the median OS and PFS were comparable between patients receiving docetaxel and those receiving paclitaxel, although these differences between docetaxel and paclitaxel were not statistically evaluated. The observed comparable PFS with a difference in ORR could be attributed to the difference in patient characteristics, such as ECOG PS 2 and liver metastasis, between patients receiving paclitaxel or docetaxel. Interpretation of the difference between the results in the previous study of paclitaxel and docetaxel [16] and in the present study may be limited due to the small number of patients.

Consistent with the present findings, several retrospective studies have demonstrated a favorable efficacy of cytotoxic chemotherapy after discontinuation of ICIs in multiple tumors. In patients with non-small cell lung cancer, the ORR of chemotherapy administered before and after ICI therapy was 35% and 53%, respectively [12]. In metastatic gastric cancer, patients receiving chemotherapy with prior exposure to ICIs achieved an ORR of 31%, whereas those receiving third-line chemotherapy without prior exposure to ICIs showed an ORR of 10% [10]. Another study for advanced gastric cancer also demonstrated that the ORR to chemotherapy was 45% in patients who previously received anti-PD-1 therapy compared to 20% in patients without [17]. In recurrent/metastatic squamous cell carcinoma of the head and neck, salvage chemotherapy following ICIs achieved an ORR of 30% [13]. Our results have expanded the current knowledge on esophageal squamous cell carcinoma.

Taxanes prevent microtubule dynamic instability, leading to inhibition of the cell cycle. In addition, paclitaxel and docetaxel have shown to reduce immunosuppressive regulatory T cells and myeloid-derived suppressor cells possibly by inducing their apoptosis [18–22]. These taxane activities may be enhanced by the residual efficacy of the preceding nivolumab treatment since binding of nivolumab on T cells was detected in peripheral blood samples more than 20 weeks after the last administration [23], and some patients exhibited long-term clinical response to nivolumab even after discontinuation of nivolumab treatment [24]. The median time from nivolumab discontinuation to the start of the subsequent taxane therapy was 0.8 months in the present study, suggesting that the remnant nivolumab may concurrently contribute to the immune response, although the effect could not be evaluated in detail owing to the lack of data on pharmacokinetics and immune cell status. Recently, the efficacy of the combination of an ICI, nivolumab and pembrolizumab, with chemotherapy as the first-line therapy for advanced esophageal cancer has been demonstrated in CheckMate 648 and KEYNOTE-590 phase III studies [25].

The incidence of any grade and grade 3–4 TRAEs observed in the present study (87% and 37%, respectively) was comparable to or even lower than that (approximately 80–100% for any grade and 50–90% for grade 3–4) in previous reports [6–8, 16]. No new safety concerns were raised in the present study.

The present study, however, has several limitations. The available information was limited to the chart of each patient, which made additional assessments impossible. The patients involved in the present study were those eligible for a clinical study; therefore, the results of the present study may not be applicable to a broad range of patients in daily clinical practice. In addition, the patients analyzed in this study, who received third-line taxane treatment after second-line nivolumab treatment, account for only 59% of the overall population in the ATTRACTION-3 study. This may have led to a selection bias for individuals with better outcomes. Finally, tumor responses were assessed by individual physicians.

## Conclusion

Favorable effectiveness and safety profile of taxanes following second-line nivolumab was observed in Japanese patients with advanced esophageal squamous cell carcinoma. When a patient with advanced esophageal cancer receiving nivolumab becomes refractory or intolerant, subsequent taxane treatment may be a promising option.

## Supplementary Information

Below is the link to the electronic supplementary material.

Supplementary file1 (DOCX 85 KB)

## Data Availability

Qualified researchers may request Ono Pharmaceutical Co., Ltd. to disclose individual patient-level data from clinical studies through the following website: https://www.clinicalstudydatarequest.com/. For more information on the policy of Ono Pharmaceutical Co., Ltd. for the Disclosure of Clinical Study Data, please visit https://www.ono.co.jp/eng/rd/policy.html.
