# Supplementary material for: Effectiveness of taxanes following nivolumab in patients with advanced esophageal squamous cell carcinoma: a retrospective chart review of patients in ATTRACTION-3
Source: Esophagus. 2022 Dec 23;20(2):302–8. doi: 10.1007/s10388-022-00972-z (PMC10024664; doi:10.1007/s10388-022-00972-z)
Supplement: Supplementary file 1 — Supplementary file1 (DOCX 85 KB) [file 10388_2022_972_MOESM1_ESM.docx]

***Esophagus***

**Supplementary material**

**Effectiveness of taxanes following nivolumab in patients with advanced esophageal squamous cell carcinoma: a retrospective chart review of patients in ATTRACTION-3**

Keisho Chin, Shun Yamamoto, Masanobu Takahashi, Shigenori Kadowaki, Yutaro Kubota, Yusuke Amanuma, Morihito Okada, Mitsuro Kanda, Yasue Kimura, Yuhiko Nogi, Yuko Arimitsu, and Yuko Kitagawa

**Corresponding author**

Keisho Chin

Department of Gastroenterological Chemotherapy

Cancer Institute Hospital

Japanese Foundation for Cancer Research

3-8-31, Ariake, Koto, Tokyo 135-8550, Japan

Email: kchin@jfcr.or.jp

**Supplementary Figure 1 Treatment outcome**

**
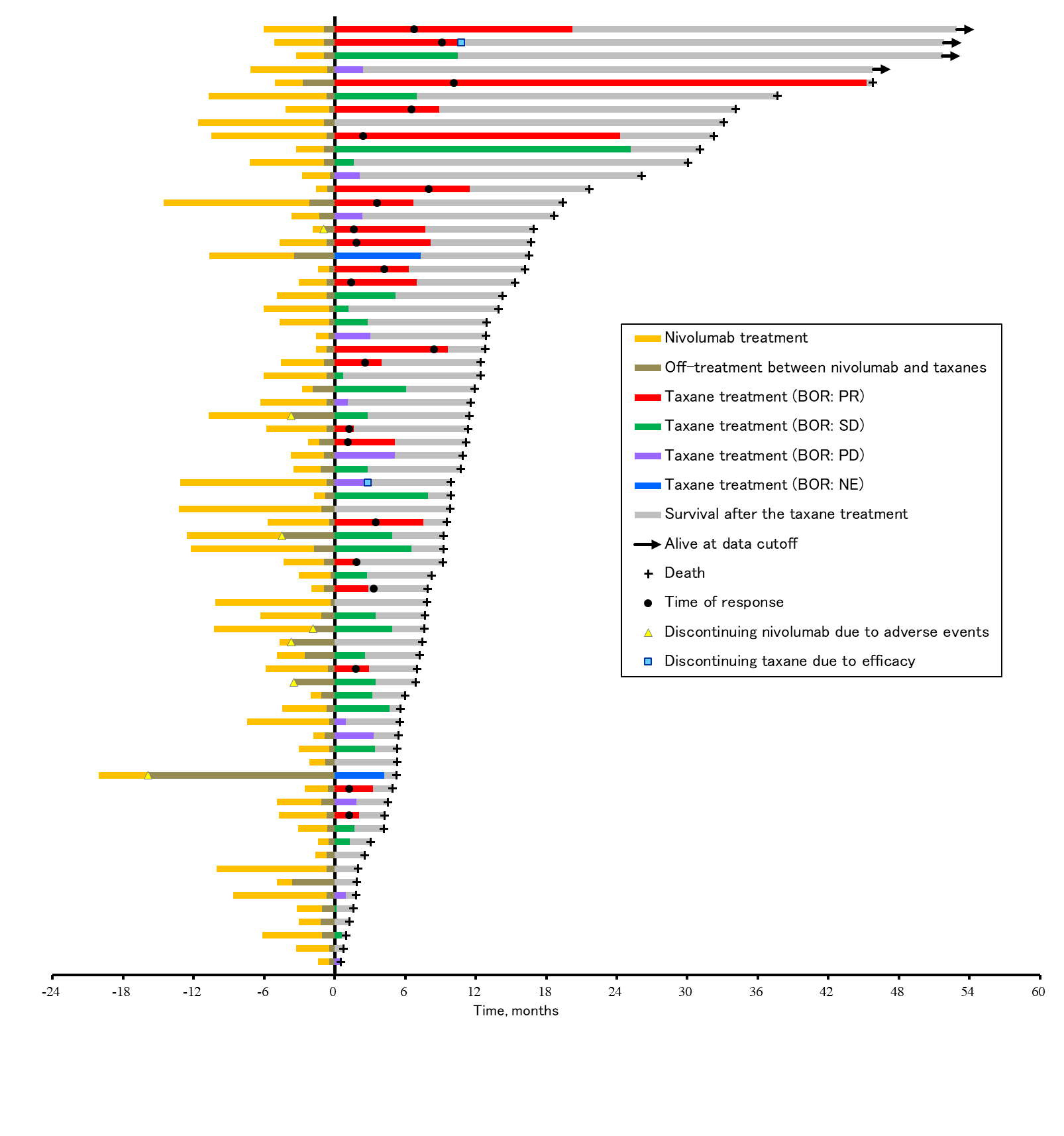
**

**Supplementary Table 1 Patient characteristics per treatment regimens**

| **Characteristics** | **Paclitaxel**  **(n = 54)**  **n (%)** | **Docetaxel**  **(n = 18)**  **n (%)** | **DCF**  **(n = 3)**  **n (%)** |
| --- | --- | --- | --- |
| Age 65 years or older | 36 (67) | 4 (22) | 1 (33) |
| Men | 43 (80) | 15 (83) | 3 (100) |
| ECOG PS |  |  |  |
| 0 | 24 (44) | 5 (28) | 3 (100) |
| 1 | 22 (41) | 10 (56) | 0 |
| 2 or higher | 7 (13) | 0 | 0 |
| Unknown | 1 (2) | 3 (17) | 0 |
| Primary lesion |  |  |  |
| Yes | 29 (54) | 11 (61) | 2 (67) |
| No | 21 (39) | 6 (33) | 1 (33) |
| Unknown | 4 (7) | 1 (6) | 0 |
| Lymph node metastasis |  |  |  |
| Yes | 44 (81) | 13 (72) | 1 (33) |
| No | 9 (17) | 4 (22) | 2 (67) |
| Unknown | 1 (2) | 1 (6) | 0 |
| Number of lymph node metastases |  |  |  |
| 1 | 11 (20) | 2 (11) | 0 |
| 2–4 | 12 (22) | 4 (22) | 1 (33) |
| 5 or more | 19 (35) | 7 (39) | 0 |
| Unknown | 2 (4) | 0 | 0 |
| Liver metastasis | 18 (33) | 2 (11) | 0 |
| Lung metastasis | 22 (41) | 8 (44) | 2 (67) |
| Best overall response to nivolumab therapy^a^ |  |  |  |
| Complete response | 0 | 0 | 0 |
| Partial response | 11 (21) | 4 (24) | 0 |
| Stable disease | 19 (37) | 8 (47) | 2 (67) |
| Progressive disease | 22 (42) | 4 (24) | 1 (33) |
| Not evaluable | 0 | 1 (6) | 0 |
| Reason for nivolumab discontinuation |  |  |  |
| Progression of primary disease | 48 (89) | 14 (78) | 3 (100) |
| Adverse events | 4 (7) | 3 (17) | 0 |
| Effectiveness | 2 (4) | 0 | 0 |
| Others | 0 | 1 (6) | 0 |

^a^A total of 72 patients had target lesion measurements before the nivolumab therapy.

DCF, docetaxel, cisplatin, and 5-fluorouracil; ECOG PS, Eastern Cooperative Oncology Group performance status.

**Supplementary Table 2 Logistic regression analysis for ORR**

**ORR Univariate analysis**

**Factors n % 95% CI Odds ratio 95% CI**

Age

<65 years 33 21.2 9.0–38.9 (reference)

≥65 years 38 36.8 21.8–54.0 2.17 0.75–6.28

Liver metastasis

No 52 26.9 15.6–41.0 (reference)

Yes 19 36.8 16.3–61.6 1.58 0.52–4.83

Lung metastasis

No 39 30.8 17.0–47.6 (reference)

Yes 31 29.0 14.2–48.0 0.92 0.33–2.58

Bone metastasis

No 60 28.3 17.5–41.4 (reference)

Yes 11 36.4 10.9–69.2 1.45 0.37–5.58

Lymph node metastasis

No 14 42.9 17.7–71.1 (reference)

Yes 57 26.3 15.5–39.7 0.48 0.14–1.60

Number of metastatic organs

0 0 NA NA

1–2 44 31.8 13.9–54.9 (reference)

≥3 18 27.8 9.7–53.5 0.82 0.25–2.77

Unknown 9 22.2 2.8–60.0 0.61 0.11–3.33

Number of metastatic sites

0 0 NA NA

1–4 22 31.8 13.9–54.9 (reference)

≥5 38 31.6 17.5–48.7 0.99 0.32–3.06

Unknown 11 18.2 2.3–51.8 0.48 0.08–2.81

ECOG PS

0 31 41.9 24.5–60.9 NA^a^

1 30 26.7 12.3–45.9 NA^a^

≥2 6 0.0 0.0–45.9 NA^a^

Reasons for nivolumab discontinuation

Disease progression 61 32.8 21.3–46.0 NA^a^

Adverse events 7 14.3 0.4–57.9 NA^a^

Others 3 0.0 0.0–70.8 NA^a^

Response to nivolumab

CR/PR 15 33.3 11.8–61.6 (reference)

SD/PD 53 28.3 16.8–42.4 0.79 0.23–2.70

Disease control in nivolumab treatment

CR/PR/SD 43 27.9 15.3–43.7 (reference)

PD 25 32.0 14.9–53.5 1.22 0.42–3.55

Duration of nivolumab treatment

<Median 32 28.1 13.7–46.7 0.88 0.32–2.46

≥Median 39 30.8 17.0–47.6 (reference)

Duration from nivolumab discontinuation to the start of taxanes

<Median 34 35.3 19.7–53.5 (reference)

≥Median 37 24.3 11.8–41.2 0.59 0.21–1.65

Radiation therapy between nivolumab discontinuation and taxanes

No 66 30.3 19.6–42.9 1.74 0.18–16.56

Yes 5 20.0 0.5–71.6 (reference)

CR, complete response; ECOG PS, Eastern Cooperative Oncology Group performance status; NA, not applicable; ORR, overall response rate; PD, progressive disease; PR, partial response; SD, stable disease

^a^ Odds ratio was not calculated because of quasi-complete separation

**Supplementary Table 3** **COX regression analysis for OS**

**OS, months Univariate analysis Multivariate analysis**

**Factors n median 95% CI HR 95% CI adjusted HR 95% CI**

Age

<65 years 34 9.6 6.0–12.9 (reference) ND

≥65 years 41 9.9 7.0–12.5 0.90 0.56–1.44 ND

Liver metastasis

No 53 9.9 7.9–12.4 (reference) (reference)

Yes 20 5.5 2.6–14.0 1.22 0.71–2.07 0.96 0.46–2.04

Lung metastasis

No 40 8.1 5.6–9.9 (reference) (reference)

Yes 32 12.0 6.9–16.6 0.78 0.48–1.26 0.46 0.23–0.94 *

Bone metastasis

No 62 10.4 7.9–12.5 (reference) ND

Yes 11 5.4 1.9–10.7 1.59 0.81–3.12 ND

Lymph node metastasis

No 15 12.0 2.6–17.0 (reference) (reference)

Yes 58 9.3 7.5–11.4 1.41 0.77–2.59 0.59 0.25–1.41

Number of metastatic organs

0 0 NA NA NA

1–2 45 9.9 7.7–12.0 (reference) (reference)

≥3 19 7.3 3.1–12.8 1.44 0.83–2.49 2.09 0.86–5.06

Unknown 11 14.3 5.6–32.3 0.70 0.35–1.41 0.03 0.00–0.29 *

Number of metastatic sites

0 0 NA NA NA

1–4 23 12.0 8.3–17.0 (reference) (reference)

≥5 39 7.7 5.4–10.9 2.07 1.19–3.61 * 2.46 1.17–5.18 *

Unknown 13 7.9 5.3–31.1 1.16 0.56–2.38 44.72 5.80–344.70 *

ECOG PS

0 32 11.5 7.9–16.2 (reference) (reference)

1 32 9.6 5.4–12.9 1.54 0.92–2.58 1.41 0.70–2.84

≥2 7 2.6 0.8–6.0 7.27 2.93–18.05 * 5.63 1.96–16.19 *

Reasons for the nivolumab discontinuation

Disease progression 65 9.9 7.7–12.5 (reference) ND

Adverse events 7 7.7 5.3–11.5 1.60 0.72–3.56 ND

Others 3 9.9 0.6–16.6 1.47 0.46–4.73 ND

Response to nivolumab

CR/PR 15 7.7 2.0–16.6 (reference) (reference)

SD/PD 56 10.8 7.9–12.4 0.89 0.50–1.58 0.72 0.28–1.84

Disease control in the nivolumab treatment

CR/PR/SD 44 9.9 7.7–12.4 (reference) (reference)

PD 27 8.3 3.1–14.0 1.09 0.67–1.80 1.03 0.38–2.77

Duration of the nivolumab treatment

<Median 36 7.7 5.4–12.0 1.35 0.85–2.16 1.65 0.70–3.92

≥Median 39 10.9 7.9–12.9 (reference) (reference)

Duration from the nivolumab discontinuation to the start of taxanes

<Median 36 9.8 5.4–12.9 (reference) ND

≥Median 39 9.9 7.5–12.0 0.83 0.52–1.33 ND

Radiation therapy between the nivolumab discontinuation and taxanes

No 69 9.9 7.7–12.4 0.56 0.24–1.30 ND

Yes 6 6.2 1.0–19.5 (reference) ND

*p<0.05

CR, complete response; ECOG PS, Eastern Cooperative Oncology Group performance status; HR, hazard ratio; NA, not applicable; ND, not determined; OS, overall survival; PD, progressive disease; PR, partial response; SD, stable disease

**Supplementary Table 4 Comparing baseline characteristics between patients with and without lung metastasis**

**Without lung metastasis^a^ With lung metastasis^a^**

**(n = 40) (n = 32)**

**Factors n (%) n (%) *P*-value**

Previous surgery^b^

No 12 (30) 19 (59) 0.0124^f^

Yes 28 (70) 13 (41)

Previous radiotherapy^b^

No 15 (38) 8 (25) 0.2583^f^

Yes 25 (63) 24 (75)

Details of radiotherapy

Curative radiotherapy 22 (55) 15 (47) 0.4931^f^

Palliative radiotherapy 3 (8) 9 (28) 0.0196^f^

Lymph node metastasis^c^

No 6 (15) 13 (41) 0.0142^f^

Yes 34 (85) 19 (59)

Unknown 0 0

Number of lymph node metastases^d^

1 9 (23) 6 (19) 0.0359^g^

2 7 (18) 3 (9)

3 1 (3) 3 (9)

4 2 (5) 0

≥5 15 (38) 6 (19)

Number of metastatic organs^c,e^

0 0 0 0.0148^g^

1–2 31 (78) 14 (44)

≥3 4 (10) 15 (47)

Unknown 5 (13) 3 (9)

Only factors with statistically significant difference between two groups were shown.

^a^At the start of taxane treatment

^b^At the start of nivolumab treatment

^c^Category "Unknown" was included in the statistical test

^d^Categories "0" and "unknown" were included in the statistical test

^e^Total number of metastatic organs including lungs, liver, bones, brain, and lymph nodes at the start of taxane treatment

^f^Chi-square test

^g^Wilcoxon rank-sum test (If "Unknown" was included, the test was performed with "Unknown" as the highest score.)

**Supplementary Table 5 COX regression analysis for PFS**

**PFS, months Univariate analysis Multivariate analysis**

**Factors n median 95% CI HR 95% CI adjusted HR 95% CI**

Age

<65 years 34 3.9 2.6–6.6 (reference) ND

≥65 years 41 5.4 3.1–7.6 0.81 0.51–1.28 ND

Liver metastasis

No 53 5.8 3.4–7.7 (reference) (reference)

Yes 20 3.4 2.5–4.9 1.30 0.78–2.20 1.26 0.62–2.57

Lung metastasis

No 40 3.9 2.6–5.8 (reference) (reference)

Yes 32 5.9 2.6–8.5 0.83 0.52–1.34 0.47 0.23–0.94 *

Bone metastasis

No 62 5.1 3.3–6.6 (reference) ND

Yes 11 3.8 1.2–9.2 1.47 0.77–2.81 ND

Lymph node metastasis

No 15 2.8 2.4–8.2 (reference) (reference)

Yes 58 4.9 3.5–6.6 1.09 0.61–1.97 0.70 0.32–1.53

Number of metastatic organs

0 0 NA NA NA

1–2 45 4.5 3.3–6.3 (reference) (reference)

≥3 19 3.8 1.9–7.8 1.31 0.76–2.28 1.41 0.57–3.51

Unknown 11 6.3 0.7–24.8 0.71 0.36–1.41 0.10 0.01–0.76 *

Number of metastatic sites

0 0 NA NA NA

1–4 23 7.7 3.5–10.9 (reference) (reference)

≥5 39 3.8 2.5–5.9 2.12 1.23–3.67 * 3.10 1.52–6.32 *

Unknown 13 5.2 0.7–21.6 1.14 0.56–2.30 12.06 1.98–73.59 *

ECOG PS

0 32 6.2 3.1–8.5 (reference) (reference)

1 32 4.3 2.8–7.3 1.21 0.74–1.99 1.28 0.66–2.49

≥2 7 2.5 0.4–3.8 3.87 1.63–9.22 * 3.32 1.21–9.09 *

Reasons for the nivolumab discontinuation

Disease progression 65 4.9 3.4–7.3 (reference) ND

Adverse events 7 4.5 1.0–6.6 1.52 0.68–3.38 ND

Others 3 3.1 0.5–7.8 1.95 0.60–6.29 ND

Response to nivolumab

CR/PR 15 4.1 1.2–7.6 (reference) (reference)

SD/PD 56 4.9 3.3–6.3 1.13 0.63–2.03 1.01 0.40–2.54

Disease control in the nivolumab treatment

CR/PR/SD 44 5.5 3.3–7.6 (reference) (reference)

PD 27 3.8 2.5–6.5 1.26 0.77–2.06 1.49 0.59–3.81

Duration of the nivolumab treatment

<Median 36 3.9 2.6–6.3 1.27 0.81–2.01 0.96 0.45–2.03

≥Median 39 5.8 3.3–7.8 (reference) (reference)

Duration from the nivolumab discontinuation to the start of taxanes

<Median 36 3.5 2.6–6.3 (reference) ND

≥Median 39 5.9 4.0–7.8 0.67 0.42–1.08 ND

Radiation therapy between the nivolumab discontinuation and taxanes

No 69 4.9 3.3–7.3 0.53 0.22–1.24 ND

Yes 6 4.9 0.5–6.3 (reference) ND

*p<0.05

CR, complete response; ECOG PS, Eastern Cooperative Oncology Group performance status; HR, hazard ratio; NA, not applicable; ND, not determined; PD, progressive disease; PFS, progression-free survival; PR, partial response; SD, stable disease
